# Research on the spoilage characteristics of whole-plant corn silage inoculated with *Clostridium beijerinckii* SHZ-8

**DOI:** 10.3389/fmicb.2025.1640283

**Published:** 2025-10-21

**Authors:** Fan Yang, Dongqing Fu, Xue Yu, Jiaying Lv, Chunhui Ma

**Affiliations:** Grassland Science, College of Animal Science and Technology, Shihezi University, Shihezi, China

**Keywords:** *Clostridium beijerinckii* SHZ-8, metabolomics analysis, microbial diversity, silage, whole-plant corn

## Abstract

Maize silage serves as a crucial feed resource for ruminants, yet its quality is frequently compromised during storage by spoilage-associated microbial activity. *Clostridium* species, particularly *Clostridium beijerinckii*, are known to induce spoilage by altering fermentation pathways. This study aimed to elucidate the effects of inoculation with *C. beijerinckii* SHZ-8 on microbial succession, metabolite profiles, and fermentation quality in whole-crop maize silage throughout the spoilage process. Silage samples were prepared with and without *C. beijerinckii* SHZ-8 inoculation. Microbial community dynamics were assessed via 16S rDNA sequencing, while metabolite alterations were characterized using untargeted metabolomics. Fermentation parameters including nutrient composition, bacterial counts, and organic acid concentrations and ratios were also determined. Correlation analyses between key metabolites and core microbial taxa were conducted. Inoculation with *C. beijerinckii* SHZ-8 significantly reduced dry matter content by 5.28% (*p* < 0.01) and lactic acid bacteria counts by 54.51% (*p* < 0.01), while increasing Clostridium abundance by 3.40 log₁₀ CFU/g FW (*p* < 0.01). The dominant fermentation mode shifted from homofermentation to heterofermentation, accompanied by an 81.6% decrease in the lactate-to-acetate ratio (*p* < 0.01). D-galacturonic acid levels exhibited a strong positive correlation with *C. beijerinckii* SHZ-8 abundance (R^2^ = 0.87, *p* < 0.01), suggesting its potential as a biomarker for *Clostridium* overgrowth. Notably, octanal and D-galacturonic acid emerged as candidate biomarkers in the inoculated group, providing a basis for the development of silage quality monitoring tools. These findings offer valuable insights for improving silage management strategies, enhancing feed preservation, and advancing the sustainability of livestock production.

## 1 Introduction

Corn silage is a key component of ruminant diets, critically affecting nutrient intake, production performance, and overall health. Its nutritional quality and hygienic safety are largely determined by the succession of microbial communities and their metabolic activities during the ensiling process (Muck, 2010; Pahlow et al., 2015). Under optimal fermentation conditions, lactic acid bacteria (LAB) rapidly convert water-soluble carbohydrates (WSCs) into lactic acid, leading to a swift pH decline that suppresses the growth of undesirable microorganisms and ensures stable, long-term preservation (Kung et al., 2018; Borreani et al., 2018).

However, the activity of certain spoilage-associated bacteria, such as *Clostridium beijerinckii* SHZ-8, can disrupt this process. These organisms generate undesirable metabolites, including butyric acid, which reduce nutritional value, impair palatability, and potentially pose health risks to livestock (Dunière et al., 2017). Elucidating the ecological role of such spoilage microorganisms in silage fermentation, as well as their interactions with other microbial taxa, is therefore of considerable scientific and practical significance.

*Clostridium beijerinckii* SHZ-8, a spore-forming and strictly anaerobic bacterium, is frequently associated with silage spoilage. Its metabolic activity—particularly the production of butyric acid—is widely recognized as a hallmark of abnormal fermentation and diminished feed value (Ogunade et al., 2018; Dunière et al., 2017; Vissers et al., 2007). However, early detection of this organism remains difficult due to the complexity of microbial population dynamics and the delayed accumulation of measurable end metabolites during the ensiling process (Sigolo et al., 2023).

Traditional methods for evaluating silage spoilage rely on sensory assessment (e.g., off-odors, discoloration) and physicochemical indicators such as elevated pH, increased ammonia-N content, and accumulation of butyric acid (Kung et al., 2018). Although effective for diagnosing advanced spoilage (Dunière et al., 2013), these indicators typically show marked changes only during the mid-to-late stages of deterioration, limiting their value for early intervention. As a result, recent research has shifted toward identifying specific biological markers (biomarkers) to enable earlier detection and precision management of the fermentation process (Sigolo et al., 2023; Borreani et al., 2018). Such biomarkers may include shifts in microbial community composition, the appearance of distinctive metabolites, or the expression of functional genes closely associated with spoilage development (Ogunade et al., 2018).

Microbial community–based biomarkers have emerged as promising tools for early spoilage detection. Previous studies tracking bacterial and fungal dynamics during fermentation and aerobic deterioration have shown that shifts in the relative abundance of certain taxa can precede visible signs of spoilage (Dunière et al., 2017). Furthermore, recent multi-omics analyses indicate that a decline in Lactobacillus populations—alongside increases in *Clostridium* spp. and the accumulation of specific amino acid degradation products—may serve as reliable indicators of early-stage deterioration (Ogunade et al., 2018).

Metabolite-based biomarkers have also been widely investigated. Strong correlations have been reported between *Clostridium* activity and aerobic instability, suggesting that microbial counts may serve as predictive indicators (Dunière et al., 2017). In addition, organic acids, biogenic amines, and specific volatile organic compounds (VOCs) have been proposed as practical markers of deterioration (Sigolo et al., 2023). Amino acid degradation products and selective volatile fatty acids have further been identified as phase-specific spoilage indicators during aerobic exposure (Borreani et al., 2018). Despite these advances, inconsistencies among reported biomarkers—largely attributable to differences in feedstock, ensiling conditions, and analytical methodologies—highlight the need for broader validation, standardized thresholds, and clearly defined detection windows.

In light of the lack of standardized strategies for the early detection of *Clostridium beijerinckii* SHZ-8–associated spoilage, this study investigated the effects of *C. beijerinckii* SHZ-8 inoculation on the microbial community structure, metabolite profiles, and fermentation quality of whole-plant corn silage. By combining high-throughput 16S rDNA sequencing with untargeted metabolomics, we aimed to elucidate the microbial and metabolic shifts triggered by this spoilage organism. The findings are expected to advance the mechanistic understanding of *C. beijerinckii* SHZ-8 in silage fermentation and to provide a theoretical basis for developing early detection methods and targeted control strategies in silage systems.

## 2 Materials and methods

### 2.1 Experimental materials

The experiment was conducted using a completely randomized design with two treatments: (1) *Clostridium beijerinckii* SHZ-8 inoculation (Group I) and (2) control without inoculation (CK). Each treatment included three biological replicates, and all measurements were performed in duplicate (technical replicates) to ensure analytical accuracy. The fermentation period lasted for 60 days, with samples collected on days 1, 7, 15, 30, and 60 for fermentation characteristics, while nutritional composition, microbial diversity, and metabolomic profiling were assessed on day 60.

The *Clostridium beijerinckii* SHZ-8 strain used in this study was originally isolated and identified in our laboratory from deteriorated whole-plant maize silage through morphological, biochemical, and 16S rRNA gene sequence analyses. The strain is preserved in our laboratory culture collection at −80 °C in 20% (v/v) glycerol stocks. For inoculum preparation, a frozen glycerol stock of *C. beijerinckii* SHZ-8 was thawed at room temperature, streaked onto enriched clostridial agar plates, and incubated anaerobically at 37 °C for 24 h. A single colony was then transferred into 50 mL of enriched clostridial liquid medium and incubated anaerobically with shaking at 160 rpm and 37 °C for 18 h. Bacterial growth was monitored by measuring optical density at 600 nm (OD₆₀₀). Cells were harvested by centrifugation at 6,000 rpm for 10 min at 4 °C, washed twice, and resuspended in sterile phosphate-buffered saline (PBS, pH 7.4) to achieve a final viable cell concentration of approximately 1.0 × 10^8^ CFU/mL, as determined by plate counting on reinforced clostridial agar.

Whole-plant maize (*Zea mays* L., cultivar Jinling Silage No. 10) was grown at the Forage Experimental Station of Shihezi University (Xinjiang, China; 44°21′4″N, 85°57′35″E; altitude 420 m), in a temperate continental arid–semiarid climate with annual precipitation of 233 mm and annual sunshine duration of 2,740.6 h. Plants were harvested at the two-thirds milk line (2/3 ML) stage and chopped into 1–2 cm pieces. For the inoculated group, maize was sprayed with *C. beijerinckii* SHZ-8 suspension at 100 mL/kg fresh weight; the control group (CK) was sprayed with the same volume of sterile water. Approximately 1 kg of fresh material per replicate was packed into polyethylene silage bags (30 × 50 cm), vacuum-sealed, and stored at 26 ± 1 °C.

#### 2.1.1 Determination of nutritional components

Nutritional composition was analyzed following (Li and Li, 2013; Li et al., 2020). Fresh samples were weighed, oven-dried at 105 °C for 30 min to deactivate enzymes, then further dried at 65 °C to constant weight for dry matter (DM) determination. Dried samples were ground and passed through a 1 mm sieve. Crude protein (CP) was determined using a Kjeldahl nitrogen analyzer (K9840, Shandong Haineng Scientific Instrument Co., Ltd., Jinan, China). Neutral detergent fiber (NDF) and acid detergent fiber (ADF) were determined using an automatic fiber analyzer (ST116A, Shandong Shengtai Instrument Co., Ltd., Jinan, China). WSCs were determined using the anthrone method (Cai et al., 2013), and starch content via the enzyme hydrolysis method (Blasel et al., 2006).

#### 2.1.2 Determination of fermentation quality

Twenty grams of silage samples collected on days 1, 7, 15, 30, and 60 were mixed with 180 mL deionized water, stored at 4 °C for 24 h, and filtered through four layers of sterile cheesecloth. Filtrate pH was measured immediately using a calibrated pH meter. Organic acids (lactic, acetic, propionic, and butyric acids) were determined by high-performance liquid chromatography (HPLC) (Agilent 1,200, Shandong Jielun Technology Products Co., Ltd., Jinan, China) following Ma et al. (2013). The filtrate was centrifuged (12,000 rpm, 3 min) and supernatants filtered through 0.22 μm aqueous-phase membranes before injection. HPLC conditions: Shodex RSpak KC-811 column (Showa Denko K. K., Tokyo, Japan) (8 mm × 300 mm); mobile phase: 3 mM perchloric acid; column temperature: 50 °C; injection volume: 5 μL; detection wavelength: 210 nm; flow rate: 1 mL/min. Ammonia nitrogen (NH_3_–N) was measured by the phenol–hypochlorite colorimetric method (Kozloski et al., 2006).

### 2.2 Experimental methods

#### 2.2.1 Determination of nutritional components

The nutritional composition was determined according to the method described by (Li and Li, 2013; Liu et al., 2021). Immediately after sampling, the fresh weight of the samples recorded. The samples were then dried in a forced-air oven at 105 °C for 30 min deactivate enzymes, followed by further drying at 65 °C until a constant weight wasachieved. DM content was subsequently calculated. DM content was measured by drying fresh whole-plant maize and silage samples at 65 °C for 72 h. After grinding and sieving the samples through a 1 mm screen, CP content was analyzed using a Kjeldahl nitrogen analyzer (K9840, Shandong Haineng Scientific Instrument Co., Ltd., Jinan, China). NDF and ADF contents were measured using an ST116A fiber analyzer (Shandong Shengtai Instrument Co., Ltd., Jinan, China). WSC were determined using the anthrone reagent method (Cai et al., 2013), and starch content was measured by the dual-enzyme hydrolysis method (Chen et al., 2016).

#### 2.2.2 Determination of fermentation quality

20 g of whole-plant corn silage samples collected on fermentation days 1, 7, 15, 30, and 60 were mixed with 180 mL deionized water, refrigerated at 4 °C for 24 h, and filtered through four layers of sterile gauze. The pH of the resulting filtrate was measured immediately using a pH meter. A portion of the filtrate was used to determine the concentrations of lactic acid, acetic acid, propionic acid, and butyric acid using a HPLC (Agilent 1,200, Shandong Jielun Technology Products Co., Ltd., Jinan, China). The filtrate was centrifuged at 12,000 rpm for 3 min, and the supernatant was filtered through an aqueous-phase filter membrane before HPLC analysis. The HPLC conditions were as follows: chromatographic column: Shodex RSpak KC-811 column (Showa Denko K. K., Tokyo, Japan) (8 mm × 300 mm); mobile phase: 3 mol/L perchloric acid solution, filtered and degassed; column temperature: 50 °C; injection volume; 5 μL; detection wavelength: 210 nm; flow rate: 1 mL/min. Another portion of the filtrate was used to measure ammonia nitrogen content using the phenol-sodium hypochlorite colorimetric method (Kozloski et al., 2006).

## 3 Microbiological indicators

### 3.1 Determination of microbial viable counts

Twenty grams of fresh whole-plant maize (raw and ensiled) were added to 180 mL of sterile physiological saline and shaken in a shaking incubator (B7 Bo’aosi General Shaking Incubator, Shanghai, China) at 120 rpm for 2 h at 37 °C. The mixture was then allowed to stand. One milliliter of the supernatant was transferred to a test tube containing 9 mL of sterile physiological saline and thoroughly mixed. Serial dilutions were prepared using sterile physiological saline.

A 100 μL aliquot of the 10^−6^ and 10^−7^ dilutions was spread onto MRS agar, malt extract agar, nutrient agar (NA), and mold medium (all purchased from Qingdao Haibo Biotechnology Co., Ltd., Qingdao, China), and incubated in an inverted position at 37 °C for 48–72 h. Simultaneously, 100 μL of the same dilutions were spread onto reinforced Clostridial agar medium (Qingdao Haibo Biotechnology Co., Ltd., Qingdao, China), placed in anaerobic gas packs (Mitsubishi Gas Chemical Company, Inc., Tokyo, Japan) with CO2 gas generators (Mitsubishi Gas Chemical Company, Inc., Japan), and incubated in an inverted position in a GH4500 water-jacketed incubator (Tianjin Teste Instrument Co., Ltd., Tianjin, China) at 37 °C for 48–72 h. Each dilution was tested in triplicate.

Colony Counting: Colonies were counted manually. Only plates with clearly distinguishable colonies and counts between 30 and 300 were used for quantification. The number of specific microorganisms per gram of fresh matter (FM), expressed as colony-forming units (CFU), was calculated using the following formula: Microbial count (CFU/g FM) = (Number of colonies × Dilution factor × 1,000 μL)/Volume of diluted sample plated (μL).

### 3.2 Determination of microbial species diversity

A 0.5 g sample was weighed and ground in liquid nitrogen. Bacterial DNA was extracted using a bacterial genomic DNA extraction kit (DP302, Tiangen Biotech Co., Ltd., Beijing, China). DNA concentration and purity were assessed using a micro nucleic acid quantifier (HM-CWF1, Shandong Hengmei Electronic Technology Co., Ltd., Weifang, China). The nucleic acid concentration was required to exceed 10 ng/μL, with an optimal 260/280 absorbance ratio between 1.8 and 2.0. Qualified DNA samples were used for PCR amplification with universal bacterial primers 27F (5′-AGAGTTTGATCCTGGCTCAG-3′) and 1492R (5′-ACGGTTACCTTGTTACGACTT-3′) (Liu et al., 2024). The PCR reaction mixture contained: 12.5 μL of 2 × Taq Platinum PCR MasterMix, 1 μL of 10 μM forward primer (F), 1 μL of 10 μM reverse primer (R), 10 μL of DNA template (approximately 50–408 ng), and 1.5 μL of ddH2O. The PCR program was as follows: initial denaturation at 94 °C for 2 min; 30 cycles of denaturation at 94 °C for 30 s, annealing at 55 °C for 30 s, and extension at 72 °C for 1.5 min; followed by a final extension at 72 °C for 2 min. The remaining amplification product was stored at −80 °C. PCR products that passed electrophoresis were sent to Sangon Biotech Co., Ltd. (Shanghai, China) for sequencing (Chen et al., 2022).

### 3.3 Determination of metabolites

Silage samples were oven-dried at 65 °C for 72 h to a constant weight, then ground and sieved to obtain powder with a particle size of <1 mm. Volatile compounds were extracted from the silage samples using a solvent extraction method (e.g., dichloromethane or ethanol). The solvent was removed under reduced pressure using a rotary evaporator, and the extract was concentrated for subsequent analysis.

An aliquot (1–2 μL) of the concentrated extract was injected into a gas chromatography (GC) system equipped with an autosampler in split/splitless inlet mode. GC parameters were as follows: column—polar or non-polar capillary GC column (e.g., DB-5 or DB-35); carrier gas—helium or nitrogen at a flow rate of 1–2 mL/min; oven temperature program—initial temperature 60 °C (1 min hold), ramp to 300 °C at 10 °C/min, final hold for 10 min.

Mass spectrometry (MS) was performed under electron impact (EI) ionization mode, coupled with time-of-flight MS to obtain high-resolution mass spectra (Raut, 2023). Data acquisition was conducted over an m/z range of 50–500, and qualitative and quantitative analyses were based on ion peak data (Liu et al., 2020).

## 4 Data processing

Nutritional composition, fermentation quality, and viable microbial counts were analyzed using both one-way analyses of variance in SPSS software, version 20.0 (IBM Corp., Armonk, NY, USA). Additional statistical analyses were performed using the stats package (version 4.3.2) in R software (version 4.3.2; R Core Team, Vienna, Austria), and data visualization was conducted with the ggplot2 package (version 3.5.0; Wickham, 2016).

## 5 Results

### 5.1 Effects of *Clostridium beijerinckii* SHZ-8 inoculation on nutrient components in whole-plant corn silage

The nutrient composition changes of whole-plant corn silage due to *Clostridium beijerinckii* SHZ-8 inoculation are detailed in Table 1. Compared to the CK group, DM content in Group I was significantly reduced by 5.28% (*p* < 0.01). Regarding WSC content, Group I was 40% lower than the CK group (*p* > 0.05).

**Table 1:** Effects of *Clostridium beijerinckii* SHZ-8 inoculation on the nutrient composition of whole-plant maize silage.

Items	CK (Mean ± SD)	I (Mean ± SD)
Dry matter (DM, g/kg FW)	29.93 ± 0.35^A^	28.35 ± 0.24^B^
Neutral detergent fiber (NDF, g/kg DM)	40.63 ± 0.12	38.49 ± 0.25
Acid detergent fiber (ADF, g/kg DM)	22.03 ± 0.23	22.75 ± 0.36
Starch (g/kg DM)	25.16 ± 0.24	20.97 ± 0.21
Crude protein (CP, g/kg DM)	8.34 ± 0.41	8.34 ± 0.25
Water-soluble carbohydrates (WSC, g/kg DM)	1.49 ± 0.12	0.90 ± 0.01

CK, Control group; I, Inoculated with Clostridium beijerinckii SHZ-8 group. For each item, values within the same row with different uppercase letters indicate extremely significant differences (*p* < 0.01). SD, Standard Deviation 528.

### 5.2 Effects of *Clostridium beijerinckii* SHZ-8 inoculation on fermentation components in whole-plant corn silage

Table 2 summarizes the effects of *Clostridium beijerinckii* SHZ-8 inoculation on the fermentation quality of whole-plant corn silage. Compared with the CK group, Group I showed a significantly higher pH (4.03 vs. 3.85, *p* < 0.01), alongside a pronounced reduction in lactic acid content by 43.8% (4.47% DM vs. 7.96% DM, *p* < 0.01). In contrast, acetic acid concentration increased by 205% in Group I (1.83% DM vs. 0.60% DM, *p* < 0.01), while butyric acid content rose markedly, showing a 2.00-fold elevation (2.14% DM vs. 0.14% DM, *p* < 0.01). The lactic acid/acetic acid ratio in Group I was 2.44, representing an 81.6% relative decrease compared with the CK group (13.17, *p* < 0.01), indicating a clear shift in fermentation pattern from homofermentative to heterofermentative metabolism.

**Table 2:** Effects of *Clostridium beijerinckii* SHZ-8 inoculation on the fermentation quality of whole-plant maize silage.

Items	CK (Mean ± SD)	I (Mean ± SD)
pH	3.85 ± 0.06^B^	4.03 ± 0.15^A^
Lactic acid, % DM	7.96 ± 0.05^A^	4.47 ± 0.61^B^
Acetic acid, % DM	0.60 ± 0.36^B^	1.83 ± 0.15^A^
Ethanol, % DM	0.18 ± 0.10	0.40 ± 0.10
Butanol, % DM	0.04 ± 0.01	0.08 ± 0.01
Propionic acid, % DM	0.07 ± 0.02	0.20 ± 0.10
Butyric acid, % DM	0.14 ± 0.05^B^	2.14 ± 0.05^A^
NH_3_–N, % DM	0.53 ± 0.25	0.60 ± 0.10
Lactic acid/Acetic acid	13.27 ± 1.33^A^	2.44 ± 0.25^B^

CK, Control group; I, Inoculated with Clostridium beijerinckii SHZ-8 group. For each item, values within the same row with different uppercase letters indicate extremely significant differences (*p* < 0.01). SD, Standard Deviation.

### 5.3 Effects of *Clostridium beijerinckii* SHZ-8 inoculation on microbial community structure in whole-plant corn silage

#### 5.3.1 Effects of *Clostridium beijerinckii* SHZ-8 inoculation on microbial diversity in whole-plant corn silage

**Figure 1:**
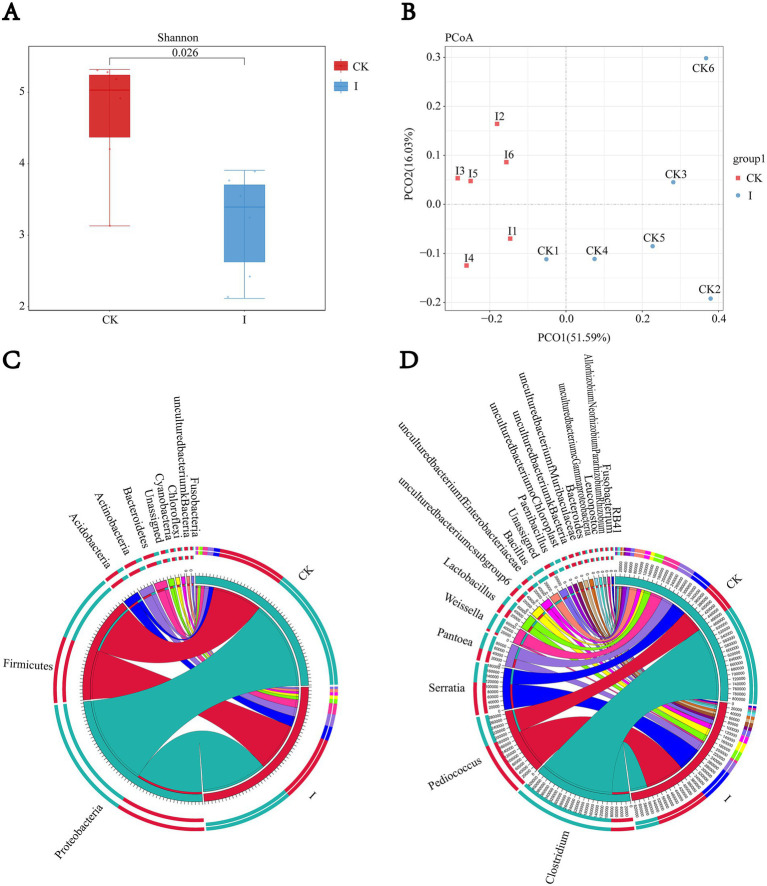
Impact of *Clostridium beijerinckii* SHZ-8 inoculation on the bacterial community structure of whole-crop maize silage. **(A)** Alpha diversity analysis showing the Shannon diversity index of bacterial communities in the control group (CK) and the *C. beijerinckii* SHZ-8 inoculated group **(I)** after 60 days of fermentation. *p*-value is indicated above the boxplots. **(B)** Principal Coordinate Analysis (PCoA) plot based on Bray–Curtis dissimilarity, illustrating the separation of bacterial communities between the control group (CK) and the *C. beijerinckii* SHZ-8 inoculated group (I). The percentage of variance explained by each principal coordinate is indicated on the axes. **(C,D)** Circos plots illustrating the relative abundance of bacterial taxa at the phylum **(C)** and genus **(D)** levels in the control group (CK) and the *C. beijerinckii* SHZ-8 inoculated group (I). The width of each segment corresponds to the relative abundance of the corresponding taxon.

The Shannon index was computed to evaluate microbial diversity, with group I exhibiting a significantly lower bacterial diversity index (3.5 vs. 5.83, *p* < 0.05) than the CK group. PCoA analysis of beta diversity between CK and group I samples demonstrated notable differences in microbial community structure (*p* < 0.05; *R*^2^ = 0.6891). Analysis at the phylum and genus levels revealed that the microbial compositions ofthe CK and group I were dominated by *Proteobacteria*, Firmicutes, and *Fusobacteria* at the phylum level. At the genus level, *Gluconobacter*, *Clostridium*, and *Pediococcus* predominated in group I, while *Pediococcus*, *Serratia*, and *Pantoea* were the dominant genera in the CK group (Figure 1).

#### 5.3.2 Effects of *Clostridium beijerinckii SHZ-8* inoculation on microbial counts in whole-plant corn silage

As shown in Table 3, group I had a significantly lower total LAB count than the CK group (2.57 vs. 5.65 log10 CFU/g FW, *p* < 0.01), representing a 54.51% reduction (*p* < 0.01). Conversely, the total clostridia count in group I was 3.40-fold higher than in the CK group (4.30 vs. 0.90 log10 CFU/g FW, *p* < 0.01). This implies that *Clostridium beijerinckii* SHZ-8 inoculation exerted a significant inhibitory effect on LAB and a promoting effect on clostridia, ultimately causing a shift in the microbial community structure of corn silage.

**Table 3:** Effects of *Clostridium beijerinckii* SHZ-8 inoculation on microbial community dynamics in whole-plant maize silage.

Microbial counts (log10 CFU/g FW)	CK (Mean ± SD)	I (Mean ± SD)
Lactic acid bacteria	5.65 ± 0.08^A^	2.57 ± 0.15^B^
Aerobic bacteria	2.44 ± 0.02	2.44 ± 0.01
Yeasts	2.30 ± 0.10	2.27 ± 0.15
Clostridium	0.90 ± 0.10^B^	4.30 ± 0.10^A^
Molds	2.19 ± 0.08	2.10 ± 0.10

CK, Control group; I, Inoculated with Clostridium beijerinckii SHZ-8 group. For each item, extremely significant differences are indicated by values in the same row with different uppercase letters (*p* < 0.01). SD, Standard Deviation.

#### 5.3.3 Changes in metabolic components in whole-plant corn silage inoculated with *clostridium beijerinckii* SHZ-8

**Figure 2:**
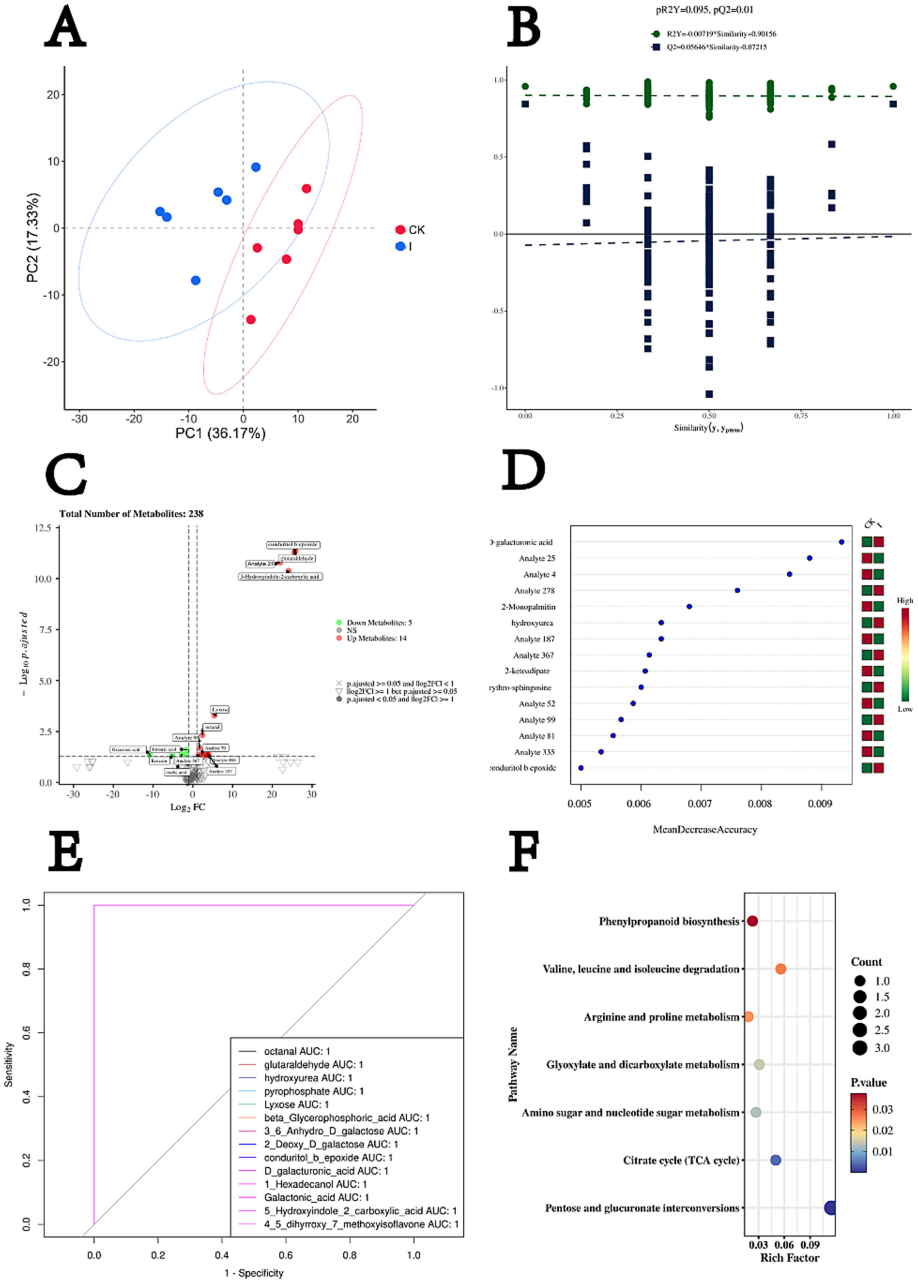
Metabolomic profiling reveals key metabolic alterations associated with *Clostridium beijerinckii* SHZ-8 inoculation in whole-crop maize silage. **(A)** PCA score plot illustrating the separation of metabolic profiles between the control group (CK) and the *C. beijerinckii* SHZ-8 inoculated group (I) after 60 days of fermentation, based on PC1 (36.17%) and PC2 (17.33%). **(B)** Permutation test results for OPLS-DA model validation, showing the distribution of *R*^2^*Y* and *Q*^2^ values from permuted datasets, with the original model’s *R*^2^*Y* and *Q*^2^ values indicated (*pR*^2^*Y* = 0.095, *pQ*^2^ = 0.01). **(C)** Volcano plot displaying differentially abundant metabolites between the *C. beijerinckii* SHZ-8 inoculated group (I) and control group (CK). Red points indicate significantly upregulated metabolites, green points indicate significantly downregulated metabolites (*p*-adjusted < 0.05 and |log2FC| > = 1). **(D)** Feature importance ranking from random forest analysis, identifying top metabolites contributing to the separation between groups based on MeanDecreaseAccuracy. D-galacturonic acid is highlighted. **(E)** Receiver ROC curves for selected metabolites, demonstrating their ability to discriminate between the *C. beijerinckii* SHZ-8 inoculated group (I) and the control group (CK) based on their AUC values. Octanal and D-galacturonic acid are highlighted with AUC = 1. **(F)** KEGG pathway enrichment analysis of differentially abundant metabolites, highlighting significantly enriched metabolic pathways. The size of the circle indicates the number of metabolites enriched in the pathway, and the color indicates the *p*-value.

Untargeted metabolomic profiling revealed clear differences between the inoculated treatment group (Group I) and the CK after 60 days of ensiling. Principal component analysis (PCA) (Figure 2A) showed distinct separation of the two groups, with the first (PC1) and second (PC2) principal components explaining 36.17 and 17.33% of the total variance, respectively. Biological replicates within each group clustered tightly, demonstrating high reproducibility of the metabolomic data. Group separation occurred primarily along PC1, indicating substantial divergence in metabolite composition. Orthogonal partial least squares discriminant analysis (OPLS-DA) further confirmed the metabolic distinction between Group I and CK (Figure 2B), yielding model parameters with strong explanatory and predictive power (*R*^2^*Y* and *Q*^2^). Model robustness was validated by 200 permutation tests, in which all permuted *R*^2^*Y* values were lower than that of the original model (all <0.9) and most permuted *Q*^2^ values fell below zero. The *p*-values for *R*^2^*Y* and *Q*^2^ were 0.095 and 0.01, respectively, confirming the statistical reliability of the model.

The primary variation was captured along the predictive component, reflecting metabolites directly associated with group classification. Volcano plot analysis (Figure 2C) identified 19 significantly altered metabolites (adjusted *p* < 0.05, |log₂FC| ≥ 1), of which 14 were upregulated and 5 were downregulated in Group I compared with CK. Notably elevated metabolites included lyxose, octanal, conduritol B epoxide, glutaraldehyde, and 5-hydroxyindole-2-carboxylic acid, whereas galactonic acid, fumaric acid, benzoic acid, and oxalic acid were significantly reduced. Random Forest classification (Figure 2D) identified D-galacturonic acid as the top discriminatory metabolite, followed by 2-monopalmitin and other features. Consistently, heatmap visualization revealed a higher relative abundance of D-galacturonic acid in Group I than in CK. Receiver operating characteristic (ROC) curve analysis (Figure 2E) further demonstrated strong discriminatory capacity, with 14 metabolites achieving perfect group classification (AUC = 1.0). Representative metabolites included octanal, xyurea, lyxose, β-glycerophosphoric acid, and D-galacturonic acid. By integrating differential metabolite profiling, Random Forest–based feature importance ranking, and ROC curve evaluation, this study identified metabolites of potential monitoring relevance. Among them, lyxose and octanal exhibited both robust statistical significance and perfect classification performance (AUC = 1.0), qualifying as potential metabolic biomarkers. In contrast, although D-galacturonic acid ranked highest in feature importance and attained an AUC of 1.0, it did not meet the statistical significance threshold and is therefore considered a candidate biomarker.

KEGG pathway enrichment analysis (Figure 2F) revealed significant metabolic divergence between Group I and CK, with seven pathways enriched (*p* < 0.05). The most prominent enrichment occurred in pentose and glucuronate interconversions (map00040; Rich factor = 0.115, count = 3, *p* = 2.10 × 10^−5^), involving D-galacturonic acid (C00181) and lyxose (C01680). The citrate cycle (TCA cycle) (map00020; Rich factor = 0.050, count = 1, *p* = 0.0042) was represented by fumaric acid (C00122). Amino sugar and nucleotide sugar metabolism (map00520; Rich factor = 0.027, count = 1, *p* = 0.0128) also included D-galacturonic acid (C00181). Glyoxylate and dicarboxylate metabolism (map00630; Rich factor = 0.031, count = 1, *p* = 0.0145) was linked to fumaric acid (C00122). Arginine and proline metabolism (map00330; Rich factor = 0.018, count = 1, *p* = 0.0253) was enriched in detected intermediates, while valine, leucine, and isoleucine degradation (map00280; Rich factor = 0.056, count = 1, *p* = 0.0268) was represented by glutaraldehyde (C00638). Additionally, phenylpropanoid biosynthesis (map00940; Rich factor = 0.023, count = 1, *p* = 0.0374) was identified as a significantly enriched pathway.

#### 5.3.4 Joint analysis of differential microorganisms and metabolites in whole-plant corn silage inoculated with *clostridium beijerinckii* SHZ-8

**Figure 3:**
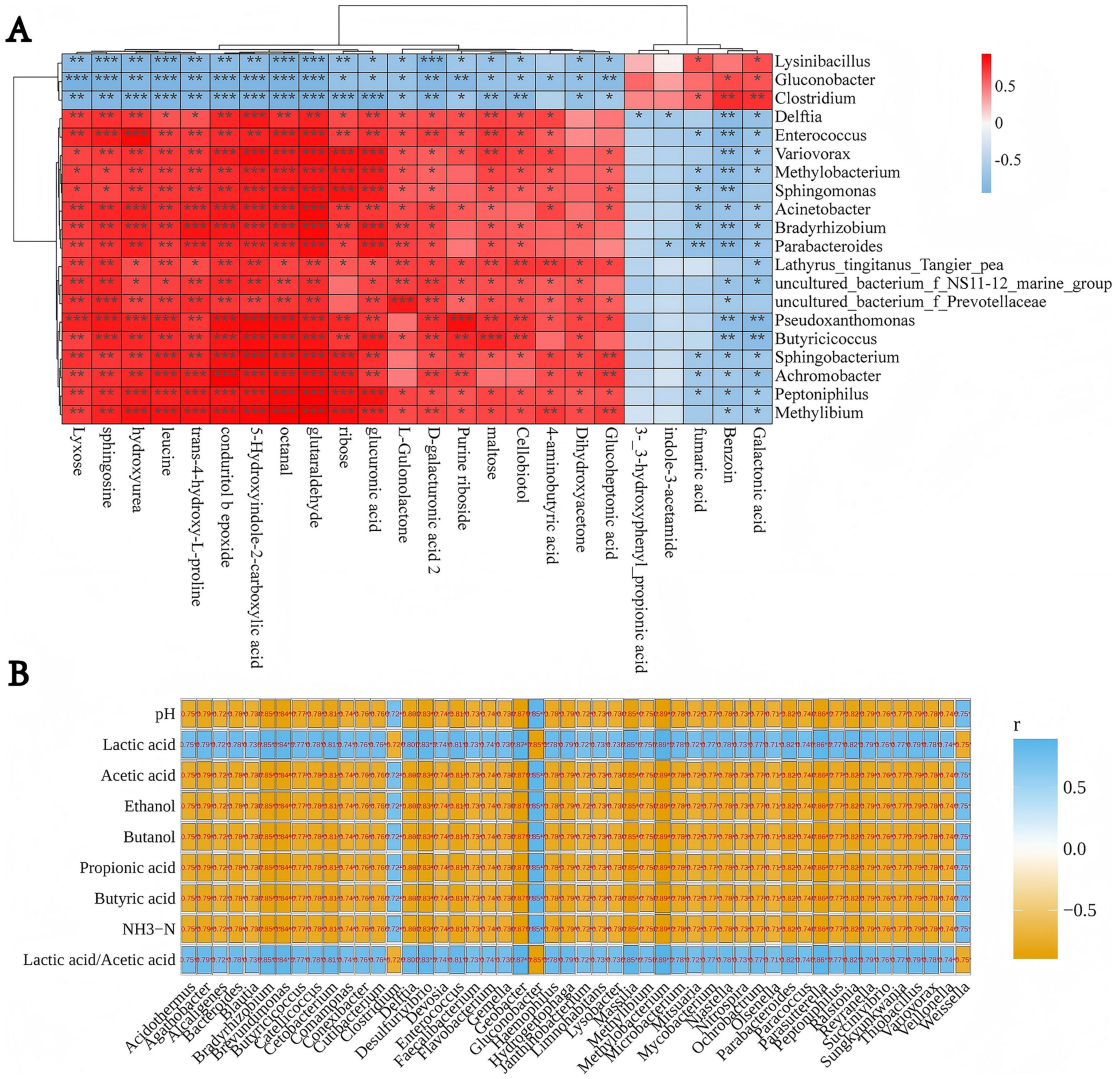
Correlation among key metabolites, microbial genera, and fermentation parameters in whole-crop maize silage inoculated with *Clostridium beijerinckii* SHZ-8. **(A)** Spearman’s heatmap shows associations between microbial relative abundance and metabolite profiles, where red denotes positive and blue denotes negative correlations; significance is indicated (*p < 0.05, **p < 0.01, ***p < 0.001). **(B)** Spearman’s heatmap presents correlations between microbial taxa and fermentation quality traits (pH, organic acids, ethanol, butanol, NH_3_–N, and lactic/acetic acid ratio), with orange for negative and blue for positive associations. Numerical values in the maps indicate correlation coefficients (*r*).

As shown in Figure 3, *Clostridium* displayed a robust positive correlation with galactaric acid (*R*^2^ = 0.87) and benzaldehyde (*R*^2^ = 0.77) (both *p* < 0.01), hinting at its metabolic role in converting complex carbohydrates into galactaric acid. It also generates aromatic compounds such as benzaldehyde through fermentative metabolic pathways. A notable positive correlation with fumaric acid (*R*^2^ = 0.67; *p* < 0.05) and negative correlations with ribose (*R*^2^ = 0.92; *p* < 0.001), leucine (*R*^2^ = 0.91; *p* < 0.001), and sphingosine (*R*^2^ = 0.88; *p* < 0.01) were identified. *Clostridium* exhibited inverse correlations with lactic acid and the lactic acid/acetic acid ratio, yet displayed positive correlations with pH (*R*^2^ = 0.75), acetic acid (*R*^2^ = 0.72), ethanol (*R*^2^ = 0.70), butanol (*R*^2^ = 0.74), propionic acid (*R*^2^ = 0.73), butyric acid (*R*^2^ = 0.72), and ammonia nitrogen (*R*^2^ = 0.75) (all *p* < 0.01). This suggests that lactic acid has an inhibitory effect on the growth of *Clostridium*; thus, as lactic acid concentration increases, the abundance or activity of *Clostridium* decreases, resulting in a negative correlation. Conversely, the increased concentrations of acetic acid, propionic acid, ethanol, butanol, and butyric acid, which are intermediate or end products of *Clostridium* metabolism, reflect an active metabolic state of *Clostridium*.

## 6 Discussion

### 6.1 Effect of *Clostridium beijerinckii* SHZ-8 inoculation on microbial community structure and quality of whole-plant corn silage

The results of this study indicated that inoculation with *Clostridium beijerinckii* SHZ-8 significantly altered the nutritional composition, fermentation quality, and metabolic end-products of whole-plant corn silage. A complex and dynamic relationship exists among microbial community succession (Zhu et al., 2025), changes in nutrient composition(Bian et al., 2022), and the production of fermentation metabolites (Zhong et al., 2018; Tian et al., 2021; Ding et al., 2023), which is influenced by both intrinsic microbial interactions and external environmental conditions, forming a multi-layered ecological network(Passos et al., 2003).

The observed reduction in LAB abundance in the SHZ-8-inoculated group can be explained by the competitive and inhibitory activities of *C. beijerinckii*. First, *C. beijerinckii* competes with LAB for available fermentable carbohydrates, particularly soluble sugars, thereby reducing the substrate pool for LAB metabolism. Second, under anaerobic conditions, *C. beijerinckii* can directly utilize lactic acid produced by LAB via secondary fermentation, converting it into acetic acid, butyric acid, CO₂, and H₂ (2 Lactic acid → Butyric acid + 2 CO₂ + 2 H₂; Lactic acid → Acetic acid + CO₂ + H₂). The consumption of lactic acid deprives LAB of its primary metabolic product, disrupting its ecological advantage. Third, the metabolic activity of *C. beijerinckii* leads to elevated silage pH due to the replacement of stronger lactic acid (pKₐ ≈ 3.8) with weaker acids such as acetic acid and butyric acid (pKₐ ≈ 4.8). This pH shift reduces the acidic stress that typically suppresses the growth of competing spoilage-associated anaerobes, while simultaneously diminishing the acid-tolerant fitness of LAB (Shu et al., 2021). Additionally, certain metabolites, including butyric acid and solventogenic by-products, may exert direct inhibitory effects on LAB growth.

Regarding metabolite accumulation, *C. beijerinckii* SHZ-8 promotes the synthesis of butyric acid, aldehydes, and alcohols through distinct but interconnected metabolic pathways. Butyric acid production primarily occurs through the acetyl-CoA–butyrate fermentation pathway, in which acetyl-CoA (derived from saccharides or from acetic acid via the reverse β-oxidation pathway) is converted to butyryl-CoA and subsequently to butyric acid. Aldehyde and alcohol formation is associated with the solventogenic phase of *C. beijerinckii* metabolism, often termed ABE (acetone–butanol–ethanol) fermentation during this process, acetyl-CoA is reduced to aldehydes by aldehyde dehydrogenase and subsequently to alcohols by alcohol dehydrogenase under reduced redox potential. For example, butyraldehyde is produced from butyryl-CoA and then reduced to butanol; similarly, acetaldehyde is reduced to ethanol. Aldehydes may also arise as transient intermediates prior to solvent conversion, and their transient accumulation, along with butanol production, is characteristic of clostridial fermentation under nutrient-limiting or redox-balanced conditions. The production of acetic acid may additionally involve the Wood–Ljungdahl pathway, enabling *C. beijerinckii* to fix CO₂ under anaerobic conditions.

Collectively, these metabolic activities lead to a decrease in lactic acid and LAB populations, while promoting the accumulation of butyric acid, aldehydes, and alcohols, resulting in elevated pH, altered organic acid profiles, and undesirable flavor compounds. These changes ultimately compromise silage quality, stability, and palatability (Lima et al., 2017; Henderson and Giddens, 1977).

### 6.2 Metabolomic and microbial correlation analyses reveal the remodeling effect of *Clostridium beijerinckii* SHZ-8 on the fermentation network of whole-crop maize silage

Metabolomic profiling combined with microbial correlation analysis revealed that *Clostridium beijerinckii* SHZ-8 exerts profound and multifaceted effects on the metabolite composition of whole-crop maize silage. Multivariate statistical analyses (PCA and OPLS-DA) demonstrated clear separation between the inoculated group (I) and the CK, highlighting the extent of metabolic reprogramming induced by Clostridium activity. These findings align with previous reports indicating that members of the genus Clostridium disrupt lactic acid–dominated fermentation by redirecting substrate fluxes toward amino acid putrefaction and alternative lipid-associated metabolic pathways (Borreani et al., 2018; Ogunade et al., 2018).

Integration of Random Forest analysis, ROC curve evaluation, and volcano plots identified a set of potential metabolic biomarkers. Both xylose and octanal emerged as core discriminative features distinguishing the inoculated group (I) from the CK, showing significant differences and perfect classification accuracy (AUC = 1.0) across statistical models, thereby qualifying as robust candidate biomarkers. Although D-galacturonic acid did not reach statistical significance in univariate analyses, it ranked highest in the Random Forest model and achieved an AUC of 1.0, underscoring its potential as a key candidate biomarker. As a functional component of plant cell walls, D-galacturonic acid may serve as an early indicator of structural degradation, providing a signal of impending spoilage before visible symptoms appear (Ochoa-Villarreal et al., 2012).

The metabolic origins of these candidate biomarkers warrant particular attention and can be broadly classified into two categories: (i) metabolites directly generated through Clostridium metabolism, and (ii) metabolites released from plant cell wall degradation. Octanal exemplifies the first category, as it is a key intermediate in the fatty acid chain-elongation and acyl-CoA reduction pathways of Clostridium metabolism (Das et al., 2020). In this study, octanal was strongly and positively correlated with *C. beijerinckii* SHZ-8 abundance in whole-crop maize silage. Its pronounced lipophilicity facilitates rapid diffusion within the silage matrix and partitioning into microbial membranes, where it disrupts membrane integrity, increases permeability, dissipates proton motive force, and induces leakage of intracellular contents. These membrane-active properties selectively suppress acid-tolerant LAB while promoting the proliferation of more resilient spoilage-associated anaerobes such as *Clostridium sporogenes* (Omoigberale, 2021). Beyond membrane disruption, octanal can react with amino groups in proteins and nucleic acids to form Schiff bases, impairing enzymatic activity and altering gene expression. Collectively, these biochemical and ecological effects accelerate the decline of beneficial microbial populations, drive community restructuring toward a clostridial-dominant state, and contribute to elevated pH, reduced palatability, and cytotoxicity at high concentrations (Kung et al., 2018). Although traditionally regarded as a transient metabolic intermediate (Hafner et al., 2013), the stable accumulation of octanal under specific fermentation conditions underscores its dual role as both a quality-deteriorating agent and a process-related biomarker of Clostridium solventogenesis.

Among the significantly upregulated metabolites, xylose was strongly linked to the hydrolysis of structural polysaccharides. Derived primarily from hemicellulose degradation, its accumulation may indicate enhanced activity of Clostridium-associated polysaccharide-degrading pathways (Weimer, 2022). However, because xylose is not exclusively produced by Clostridium (Broeker et al., 2018), its specificity as a diagnostic marker is lower than that of octanal in complex microbial communities. Accordingly, xylose is better suited as an auxiliary indicator of *Clostridium* metabolic activity rather than a standalone, specific biomarker.

D-Galacturonic acid, a direct product of homogalacturonan degradation in plant cell wall pectin, is primarily generated in silage through the activity of microbial pectinolytic enzymes—most notably pectate lyases (PL1, PL9) and polygalacturonases—which cleave the α-(1 → 4)-linked galacturonic acid backbone (Leschine, 2005). Under anaerobic conditions, such enzymes are predominantly associated with Clostridium spp. (e.g., *C. beijerinckii*) and a limited number of other microorganisms (ten Have et al., 2002). Genome annotations of *C. beijerinckii* and related clostridia have identified polysaccharide utilization loci (PULs) encoding PL1, PL9, α-L-arabinofuranosidase, and *β*-xylosidase (Huang et al., 2022; Huang et al., 2025). These enzymes act synergistically to remove arabinan and xylan side chains, expose the homogalacturonan backbone, and release both D-galacturonic acid and xylose. KEGG pathway enrichment links these activities to the “Pentose and glucuronate interconversions” pathway, which channels liberated D-galacturonic acid into uronate isomerase– and dehydrogenase-mediated reactions, producing pyruvate and glyceraldehyde-3-phosphate for fermentation metabolism (Seveso et al., 2024). Because pectin is among the earliest cell wall polymers targeted during anaerobic spoilage—and pectinolytic activity is common in clostridia but rare in LAB—D-galacturonic acid represents a highly specific early-stage signal of clostridial pectinolysis. Its release precedes extensive cellulose degradation, thereby providing early warning of structural carbohydrate loss and potential nutrient depletion. In this study, D-galacturonic acid concentration exhibited a strong positive correlation with *Clostridium* relative abundance (*R*^2^ = 0.87), reinforcing its potential as an early-warning biomarker of clostridial-driven deterioration.

Correlation analyses revealed that the observed metabolic shifts closely reflected a transition from lactic acid–dominated to butyric acid–dominated fermentation—a hallmark of *Clostridium* overgrowth. Significant positive correlations were observed among butyric acid, propionic acid, ethanol, butanol, ammonia-N, and pH, indicating a metabolic deviation from lactic acid–driven acidification toward proteolytic and solvent-producing pathways. In contrast, lactic acid concentration and the lactate/acetate ratio were significantly negatively correlated, suggesting delayed or impaired lactic acid production. This reduction weakened a critical ecological barrier against *Clostridium* expansion, thereby creating favorable conditions for *C. beijerinckii* SHZ-8 to dominate the silage microbiome. The resulting metabolic shift exerted a dual impact at ecological and biochemical levels: (i) hydrolysis of plant cell wall polysaccharides disrupted fiber structure, releasing soluble sugars that fueled *Clostridium* metabolism while depleting structural carbon reserves and causing irreversible nutrient loss; and (ii) these sugars drove the production of butyric acid, aldehydes, and alcohols, which in turn elevated pH, promoted protein degradation with ammonia-N release, and accelerated spoilage progression.

Metabolomic–microbial correlation analyses demonstrated that *Clostridium beijerinckii* SHZ-8 profoundly reconfigures the metabolic network of whole-crop maize silage, with octanal and D-galacturonic acid emerging as key indicators of fatty acid reduction and pectin degradation, respectively. These metabolites mark the transition from lactic acid–dominated fermentation to butyric acid-, alcohol-, and aldehyde-rich fermentation. This shift compromises the fermentation barrier, accelerates nutrient loss, elevates pH, and increases hygienic risks. Concurrent monitoring of these metabolites alongside conventional indicators offers a robust early-warning framework for controlling *Clostridium* overgrowth. Looking ahead, validation under farm-scale conditions—where environmental heterogeneity, forage composition, and management practices vary—will be critical. Progress will also depend on developing rapid, field-deployable assays that balance analytical specificity with the sensitivity required to detect low-abundance early-stage markers. Integrating such portable, user-friendly platforms into routine farm operations would enable real-time monitoring and precision interventions, ultimately advancing silage preservation strategies.

## 7 Conclusion

This study shows that inoculation with *Clostridium beijerinckii* SHZ-8 significantly accelerates nutrient loss and increases spoilage risk in whole-crop maize silage, characterized by marked reductions in DM content and LAB counts, extensive proliferation of *Clostridium*, and a clear shift in fermentation from homofermentation to heterofermentation. Integrated metabolomic and microbiome analyses identified galacturonic acid as an early-stage metabolic biomarker of silage deterioration. These insights establish a scientific basis and practical framework for early spoilage detection and the development of targeted preservation strategies, offering substantial value for quality control and risk management in silage production.

## Data Availability

The sequencing data generated in this study have been deposited in the NCBI Sequence Read Archive (SRA) under the BioProject accession number PRJNA1338828.
